# Real-time LC-OCT monitoring of scalp hypertrophic actinic keratosis clearance following cryotherapy, laser, and 5-fluorouracil therapy

**DOI:** 10.3389/fmed.2026.1895820

**Published:** 2026-08-26

**Authors:** Giulio Bortone, Vincenzo Coppolelli, Luca Gargano, Francesca Svara, Mehdi Boostani, Steven Paul Nisticò, Giovanni Pellacani, Carmen Cantisani

**Affiliations:** 1 UOC of Dermatology, Department of Medical and Cardiovascular Science, La Sapienza Medical School of Rome, Rome, Italy; 2 Department of Dermatology, Roswell Park Comprehensive Cancer Center, Buffalo, NY, United States

**Keywords:** 5-fluorouracil, biological response, CO_2_ laser, cryotherapy, DEJ undulation index, field cancerization, hypertrophic actinic keratosis, LC-OCT

## Abstract

**Introduction:**

Assessment of treatment response in hypertrophic actinic keratoses (hAKs) remains largely dependent on clinical examination and dermoscopy, although apparent clearance may not reflect complete resolution of imaging features associated with residual disease. Line-field confocal optical coherence tomography (LC-OCT) enables non-invasive, longitudinal visualization of epidermal, dermal-epidermal junction, and superficial dermal changes at near-cellular resolution.

**Objective:**

To characterize LC-OCT response patterns in scalp hAKs treated with ablative CO_2_ laser, cryotherapy, or topical 0.5% 5-fluorouracil/10% salicylic acid, and to evaluate the potential role of LC-OCT in identifying persistent imaging features suggestive of incomplete treatment response requiring retreatment or treatment escalation.

**Methods:**

This retrospective study included 150 scalp hAKs classified at baseline as KIN 2–3 and PRO II-III, allocated to CO_2_ laser, cryotherapy, or topical 5-FU/SA groups. LC-OCT images acquired during routine follow-up were evaluated at baseline and post-treatment time points for stratum corneum thickness, epidermal thickness, keratinocyte atypia, PRO score, dermal-epidermal junction undulation index, intercellular edema, vascular pattern, and modality-specific structural changes. Treatment response was assessed according to LC-OCT-defined imaging criteria rather than histopathological confirmation.

**Results:**

Cryotherapy produced early sub-epidermal blistering, followed by partial imaging response requiring repeated sessions in 40% of lesions. CO_2_ laser induced immediate epidermal ablation, rapid re-epithelialization, and complete response after retreatment in residual cases, with long-term LC-OCT evidence of dermal collagen remodeling. Topical 5-FU/SA showed heterogeneous responses; 65% of lesions, mainly KIN 3/PRO III, required escalation to a physical modality.

**Conclusion:**

LC-OCT enables non-invasive monitoring of treatment-related hAKs modifications and may identify persistent imaging abnormalities despite apparent clinical resolution, supporting individualized sequential treatment strategies.

## 1 Introduction

Actinic keratosis (AK) is currently regarded as a chronic and recurring *in situ* keratinocytic neoplasia arising from the clonal expansion of atypical keratinocytes within the epidermis, driven by cumulative ultraviolet radiation exposure (1–3). Basal epidermal keratinocyte atypia and the pattern of downward proliferation at the dermal-epidermal junction, rather than clinical appearance alone, appear to drive malignant transformation toward invasive squamous cell carcinoma (SCC) (4, 5). Within this framework, hypertrophic actinic keratoses (hAKs), corresponding to Olsen grade 2–3 lesions, represent a clinically and biologically distinct subset characterized by marked stratum corneum thickening, a greater degree of keratinocyte dysplasia and a higher risk of progression to invasive SCC compared to flat lesions (6–8).

The classification of AK has evolved considerably over the past two decades. The histopathological grading system proposed by Roewert-Huber distinguishes three grades based on the vertical extent of keratinocyte atypia within the epidermis (9, 10). The concept of Keratinocytic Intraepidermal Neoplasia (KIN), introduced by Cockerell, reframes AK as a continuous neoplastic spectrum with corresponding prognostic implications (11, 12). The PRO score (I to III), more recently validated by Ruini et al. (4) grades the malignant potential of AK through the degree of DEJ undulation and downward keratinocyte proliferation toward the papillary dermis, independently of the vertical extent of atypia, and has demonstrated the strongest correlation with histological risk of SCC progression among available non-invasive grading systems (4, 13–15). Collectively, these classification frameworks reflect a shared recognition that AK represents a biological continuum rather than a discrete clinical entity, and that the degree of keratinocyte dysplasia and the pattern of proliferative engagement at the dermal-epidermal junction carry independent prognostic weight in determining the risk of progression toward invasive SCC.

For the treatment of hAKs three lesion-directed methods are frequently applied: cryotherapy with liquid nitrogen, the ablative CO_2_ laser and topical 0.5% 5-fluorouracil (5-FU) combined with 10% salicylic acid (SA). Cryotherapy for hAKs destroys atypical cells in hAKs within minutes using a fast freeze-thaw process leading to cryonecrosis of the atypical cells of hAKs (16, 17, 47, 48). It is a common and relatively inexpensive procedure that can be performed in the clinic and is usually an outpatient procedure. The ablated layer of the epidermis is removed by the laser and within 6–12 months dermal collagen remodeling occurs (18, 45, 46). The typical application of 5-FU 0.5% in combination with 10% SA, the keratolytic agent, results in the inhibition of thymidylate synthase in the rapidly dividing atypical cells (19). The resulting block to DNA synthesis is temporary and once all abnormal cells have been killed, the normal surrounding epidermis returns to its usual functions (20). As with other forms of 5-FU, there is some variability in efficacy between individuals and it may require repeated courses to achieve complete clearance (21, 22). Importantly, available data indicate that all three of the established lesion-directed treatments (cryotherapy, ablative CO_2_ laser and topical 5-FU) result in clearance in approximately 70%–80% of AK cases and therefore there are no data to support the claim of superior efficacy of any of the established treatments in unselected populations of patients with AK (16, 23, 24, 44). Of importance, however, in the subset of hypertrophic AK with advanced KIN scores, there are now available data to indicate clinically relevant differences between the established treatments with respect to depth of action and the duration of remission, although these findings have yet to be systematically characterized at the level of structural and cellular pathology (25, 26). Monitoring of treatment is generally confined to clinical examination with or without dermoscopy but as with histological assessment of treated lesions, these tools are unable to detect residual atypical keratinocytes in the depths of a lesion that has clinically completely cleared (27–29). Consequently, such lesions will commonly relapse early, and there is a clear unmet clinical need for an imaging modality or tool that is able to assess the effects of treatment on a molecular and cellular level without the need for a biopsy.

Skin imaging is an emerging and rapidly expanding field in dermatology, offering non-invasive tools for diagnosis, risk stratification, treatment monitoring, and longitudinal assessment of skin cancers and precancerous lesions (30–38). Line-field confocal optical coherence tomography (LC-OCT) is a non-invasive real-time imaging modality combining the principles of reflective confocal microscopy and optical coherence tomography to generate two-dimensional vertical and horizontal images as well as three-dimensional reconstructions of the skin *in vivo*, with a lateral resolution of 1.1 μm, an axial resolution of 1.3 μm and a tissue penetration depth of approximately 500 μm (39, 40). Vertical LC-OCT sections are directly comparable to conventional histopathological sections, enabling real-time visualization of epidermal architecture, DEJ morphology and papillary dermal structure at cellular resolution (39, 40). LC-OCT criteria for AK have been characterized in previous studies and include hyperkeratosis, parakeratosis, acanthosis, keratinocytic atypia, disrupted DEJ and dilated dermal vessels. The PRO score has been shown to be non-invasively assessable by LC-OCT with a concordance with histology of up to 75% (weighted kappa 0.66), and artificial intelligence-based algorithms for automated PRO score quantification from LC-OCT images have recently been validated with comparable performance (4, 15).

Recent advances in artificial intelligence, including convolutional neural networks and multimodal large language models, have shown increasing potential for automated detection, classification, and clinical interpretation of actinic keratoses and skin cancers from dermatologic images; however, their integration with high-resolution modalities such as LC-OCT remains an emerging area requiring prospective clinical validation (41).

Despite this diagnostic potential, studies specifically addressing the role of LC-OCT in the longitudinal monitoring of hAK treatments remain limited, with existing evidence restricted to small cohorts and single-modality observations. No study to date has systematically compared the LC-OCT response profiles of the three most commonly used lesion-directed modalities across multiple time points. The present study aims to characterize modality-specific LC-OCT response patterns at each time point in a retrospective analysis of 150 hAKs treated with ablative CO_2_ laser, cryotherapy or topical 5-FU/SA, and to evaluate the potential role of LC-OCT in detecting persistent imaging abnormalities after treatment and supporting longitudinal monitoring.

## 2 Methods

### 2.1 Study design and patient selection

This study is a retrospective analysis of LC-OCT imaging data routinely acquired during standard clinical follow-up visits at the Dermatology and Venereology Unit of Sapienza University of Rome. Images were retrieved from the institutional clinical archive and selected according to predefined inclusion and exclusion criteria. No additional visits or procedures were performed for the purpose of this study; all acquisitions were part of routine clinical management.

Cases were eligible for inclusion if the patient was an adult (age 55–92 years) with a clinical and dermoscopic diagnosis of hypertrophic actinic keratosis (Olsen grade 2–3) on the scalp, and if the lesion had been classified as KIN 2 to 3 and PRO II to III by LC-OCT imaging at baseline prior to treatment. Cases were excluded if any of the following conditions were documented in the clinical record: immunosuppression of any cause, concurrent systemic treatments with known activity on AK, known allergy or contraindication to any of the study treatments, insufficient LC-OCT image quality at baseline precluding reliable DEJ visualization after optimization, or clinical or dermoscopic suspicion of invasive SCC requiring immediate biopsy.

A total of 150 hAK lesions met the selection criteria and were allocated to three groups of 50 lesions each according to the treatment received: ablative CO_2_ laser, cryotherapy with liquid nitrogen, and topical 0.5% 5-fluorouracil combined with 10% salicylic acid. Treatment allocation had been based on individualized clinical assessment of lesion grade, degree of hyperkeratosis, anatomical distribution and patient-related factors. Accordingly, the study was designed to describe treatment-specific LC-OCT response patterns observed during routine clinical practice rather than to compare the efficacy of the different therapeutic modalities.

### 2.2 Treatment protocols

Ablative CO_2_ laser treatment was performed under local anesthesia using a SmartXide fractional CO_2_ laser system (DEKA M.E.L.A. Srl, Florence, Italy). Lesions were treated with progressive ablative passes until complete removal of the hyperkeratotic and dysplastic epidermal layers, with the clinical endpoint defined as the appearance of the papillary dermis. Cryotherapy was performed using liquid nitrogen applied via spray technique, with a freeze time calibrated to lesion thickness (10–20 s per cycle) and two freeze-thaw cycles per session. Retreatment sessions were performed at 4-week intervals in lesions showing incomplete response.

Topical 0.5% 5-fluorouracil combined with 10% salicylic acid was applied once daily by the patient to the target lesion for a total of 12 weeks, in accordance with the approved prescribing information. In lesions with marked baseline hyperkeratosis, pre-treatment with 20% salicylic acid ointment under occlusion was recommended for 2 weeks prior to initiation to facilitate drug penetration.

### 2.3 LC-OCT imaging and PRO score grading

All LC-OCT acquisitions were performed using the DeepLive device (DAMAE Medical, Paris, France) with integrated dermoscopic guidance. Acquisitions included vertical B-scan sections and horizontal *en-face* sections. A minimum of three vertical sections per lesion were obtained from the most representative area identified by dermoscopy at each time point. In lesions with marked baseline hyperkeratosis, DEJ visualization was optimized prior to acquisition either by pre-treatment with 20% salicylic acid ointment or by gentle curettage of the hyperkeratotic surface immediately before imaging, both of which yielded a significant improvement in DEJ visibility on vertical LC-OCT sections.

PRO score and KIN grading were assigned as part of routine clinical evaluation. LC-OCT images were reviewed by dermatologists experienced in LC-OCT interpretation as part of routine clinical practice, in accordance with the criteria described by Ruini et al. (4) and the KIN classification proposed by Cockerell (11). Image assessment was not performed in a blinded fashion, and no formal interobserver agreement analysis was conducted, reflecting the retrospective observational design of the study. In cases where the PRO score was uncertain or borderline, grading was determined by collegial discussion among the attending dermatologists, as per standard clinical practice at our institution.

### 2.4 Follow-up time points

LC-OCT acquisitions had been performed as part of routine clinical practice at standard follow-up visits: before treatment initiation (T0), within the first 24–72 h after treatment for CO_2_ laser and cryotherapy groups (T1), at approximately 4 weeks (T2), at approximately 8 weeks (T3), and at 6–12 months (T4). These time points reflect standard clinical review intervals routinely adopted in the management of hypertrophic AK at our institution. All LC-OCT images were retrospectively retrieved and analyzed for the purposes of this study. For the topical 5-FU/SA group, T1 was not applicable; the first post-baseline LC-OCT evaluation was therefore available at the four-week visit (T2).

### 2.5 LC-OCT parameters assessed

**Figure 1:**
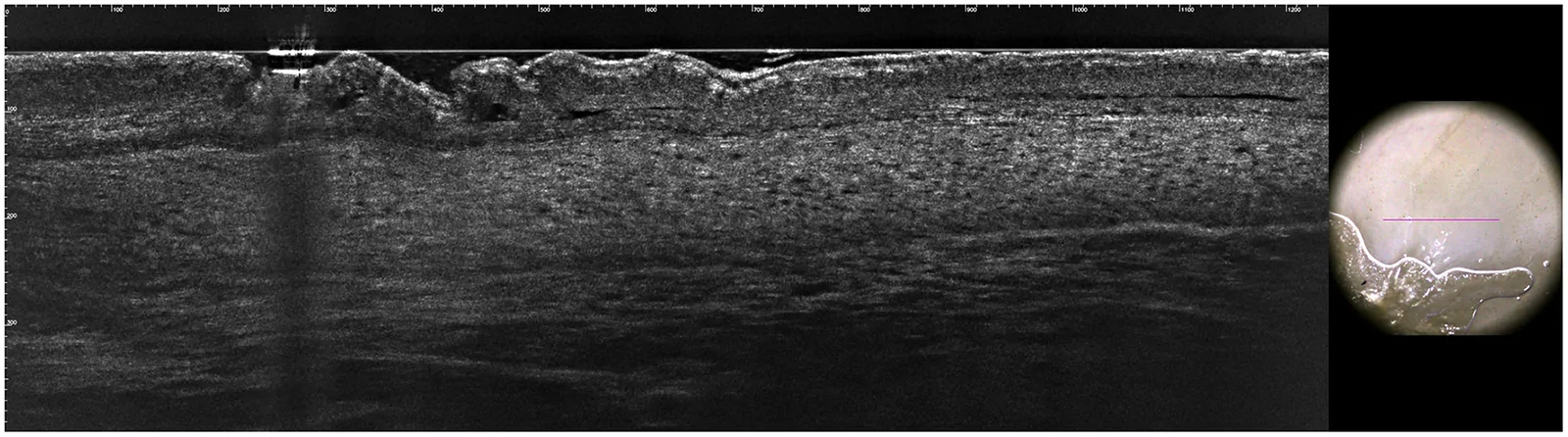
Baseline LC-OCT features of hypertrophic actinic keratosis. Representative vertical LC-OCT image of a scalp hypertrophic actinic keratosis before treatment, showing marked hyperkeratosis, an irregular and thickened epidermis, architectural disorganization consistent with keratinocyte atypia, and an undulating dermal-epidermal junction with downward epidermal protrusions. The accompanying dermoscopic image shows the corresponding hyperkeratotic lesion.

**Figure 2:**
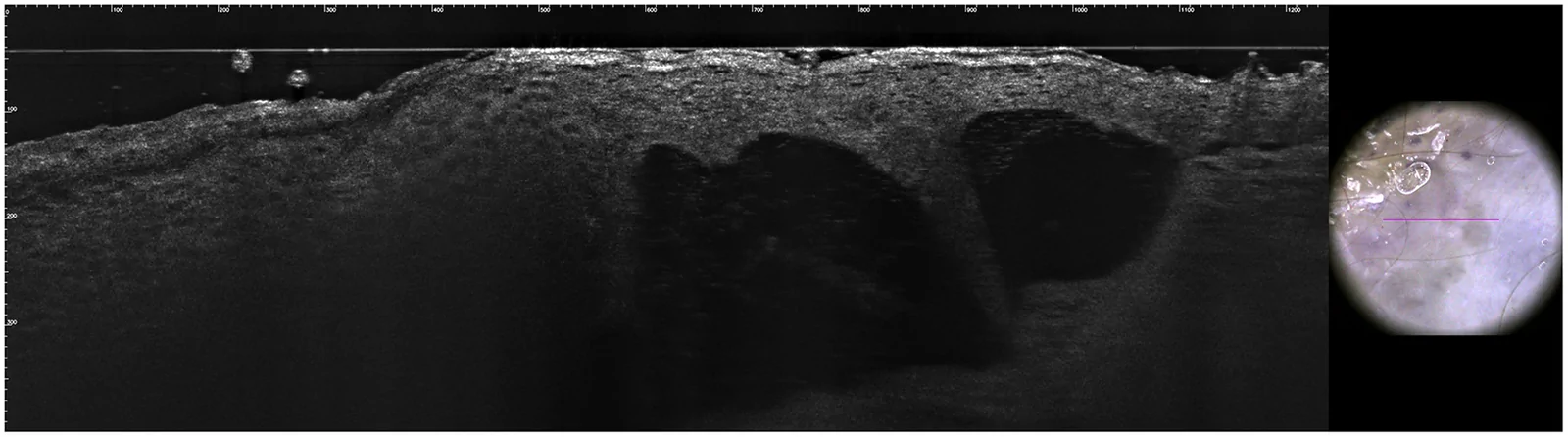
Early LC-OCT changes following cryotherapy. Representative vertical LC-OCT image obtained shortly after cryotherapy, showing prominent hyporeflective subepidermal spaces consistent with treatment-induced blister formation and dermo-epidermal separation. Fluid accumulation limits assessment of the dermal-epidermal junction. The accompanying dermoscopic image shows the corresponding treated lesion.

**Figure 3:**
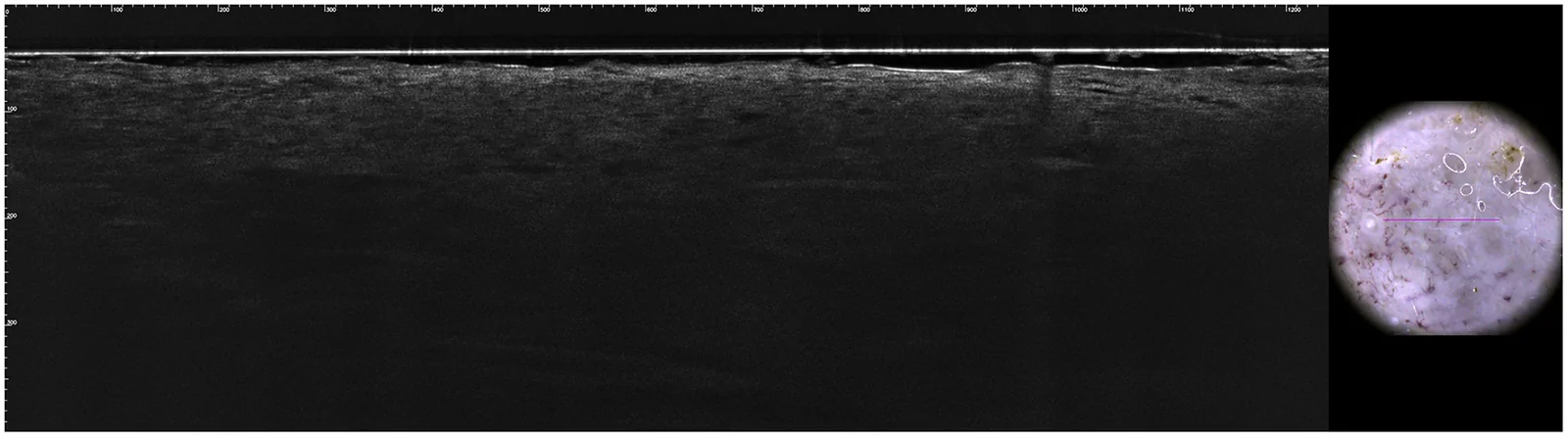
Early LC-OCT changes following ablative CO_2_ laser treatment. Representative vertical LC-OCT image obtained shortly after ablative CO_2_ laser treatment, showing removal of the hyperkeratotic and dysplastic epidermal layers, surface flattening, and exposure of the superficial dermis. The accompanying dermoscopic image demonstrates the corresponding post-treatment surface changes.

**Figure 4:**
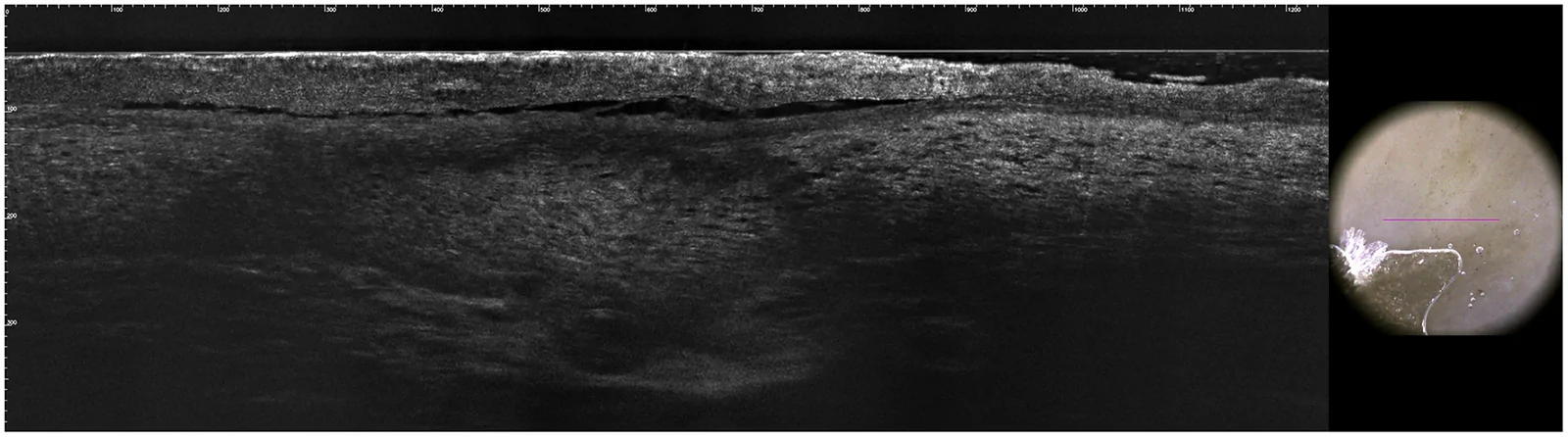
LC-OCT changes during topical 5-fluorouracil/salicylic acid treatment. Representative vertical LC-OCT image acquired during treatment with topical 0.5% 5-fluorouracil and 10% salicylic acid, showing treatment-related epidermal architectural alteration, hyporeflective intraepidermal spaces, and inflammatory changes consistent with a cytotoxic and keratolytic response. The accompanying dermoscopic image shows the corresponding treated lesion.

**Figure 5:**
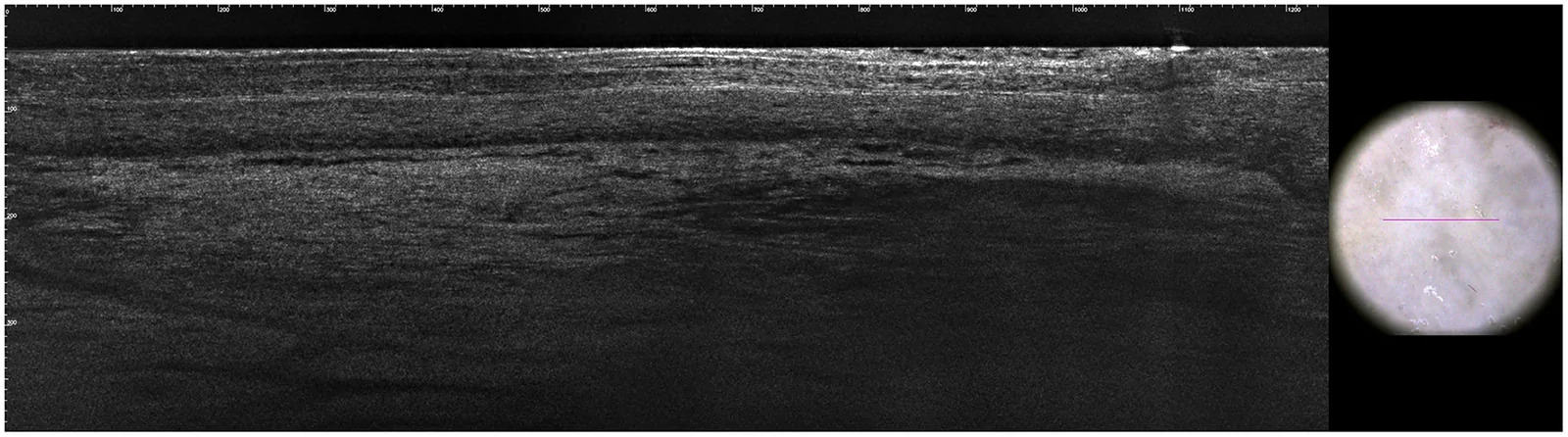
LC-OCT appearance 6 months post-treatment. Representative vertical LC-OCT image obtained 6 months post-treatment, showing a thin and regular epidermis, normalization of dermal-epidermal junction architecture, and an absence of evident residual hypertrophic actinic keratosis features. The accompanying dermoscopic image shows the corresponding clinical appearance.

The following LC-OCT parameters were systematically evaluated at each time point: stratum corneum thickness, epidermal thickness, PRO score, dermal-epidermal junction undulation index, keratinocyte atypia, intercellular edema, and dermal vascular pattern. Stratum corneum thickness was defined as the vertical extent of the hyperreflective superficial layer, whereas epidermal thickness was measured from the base of the stratum corneum to the dermal-epidermal junction. PRO score was graded as I, II, or III according to the absence of protrusions, presence of rounded protrusions, or presence of cone-shaped/spike-like deep protrusions, respectively, as described by Ruini et al. (4). The dermal-epidermal junction undulation index was calculated as the ratio between the measured DEJ contour length and the horizontal field width, expressed as a percentage; values below 15% were considered consistent with PRO I. Keratinocyte atypia was graded as absent, mild, moderate, or severe according to KIN criteria. Intercellular edema was assessed by the presence and distribution of hyporeflective spaces between adjacent keratinocytes, and dermal vascular pattern was classified as normal, mildly increased, or atypical. Representative baseline LC-OCT features are shown in Figure 1. Treatment-specific post-treatment structural changes, including cryotherapy-induced subepidermal hyporeflective space, CO_2_ laser-induced ablation and granulation tissue, topical 5-FU/SA-associated intraepidermal fluid or cystic changes, and long-term dermal collagen reorganization after CO_2_ laser, are shown in Figures 2–5.

For the purposes of this study, LC-OCT-defined complete imaging response was defined as the simultaneous normalization of the monitored LC-OCT parameters, including restoration of a regular epidermal architecture, absence of keratinocyte atypia, normalization of the dermal-epidermal junction with disappearance of PRO II/III protrusions, marked reduction or disappearance of hyperkeratosis, and absence of imaging features suggestive of persistent active disease. Lesions not fulfilling all these criteria were classified as showing incomplete LC-OCT-defined response. This imaging endpoint was used for longitudinal monitoring and should not be interpreted as histopathological confirmation of complete lesion clearance. Only descriptive statistics (counts and percentages) were used to summarize the findings; given the retrospective, non-randomized design and the unbalanced baseline characteristics across groups, no inferential statistical testing or formal between-group efficacy comparison was performed.

## 3 Results

### 3.1 Patient and lesion characteristics

A total of 100 patients (age range 55–92 years; 75 male/25 female) contributing 150 hypertrophic actinic keratoses met the selection criteria and were included in the analysis. All lesions were located on the scalp. At baseline, PRO score distribution across the entire cohort was PRO II in 60 lesions (40%) and PRO III in 90 lesions (60%); KIN grading showed KIN 2 in 85 lesions (57%) and KIN 3 in 65 lesions (43%). As expected in a non-randomized study with clinician-driven treatment allocation, the three treatment groups differed in baseline lesion characteristics: the CO_2_ laser group comprised a higher proportion of KIN 3 lesions (74%) compared to the cryotherapy (32%) and topical 5-FU/SA (24%) groups, reflecting the clinician's preference for ablative treatment in more advanced lesions. Conversely, the cryotherapy group showed the highest proportion of PRO III lesions (76%), consistent with the use of cryotherapy in hyperkeratotic lesions not selected for laser treatment. Given the marked hyperkeratosis characterizing all included lesions, gentle curettage of the hyperkeratotic surface was performed systematically prior to LC-OCT acquisition in order to ensure adequate DEJ visualization on vertical sections; in selected cases with particularly thick keratotic debris, additional pre-treatment with 20% salicylic acid ointment was applied before imaging (Table 1).

**Table 1:** KIN grading and PRO score represent independent classification systems applied to the same lesions.

Parameter	CO_2_ laser (*n* = 50)	Cryotherapy (*n* = 50)	Topical 5-FU/SA (*n* = 50)	Total (*n* = 150)
KIN 2, *n* (%)	13 (26%)	34 (68%)	38 (76%)	85 (57%)
KIN 3, *n* (%)	37 (74%)	16 (32%)	12 (24%)	65 (43%)
PRO II, *n* (%)	20 (40%)	12 (24%)	28 (56%)	60 (40%)
PRO III, *n* (%)	30 (60%)	38 (76%)	22 (44%)	90 (60%)

Each system sums to the total of 150 lesions. Baseline lesion characteristics across the three treatment groups. KIN, keratinocytic intraepidermal neoplasia; PRO, Proliferation score; 5-FU/SA, 5-fluorouracil/salicylic acid.

### 3.2 LC-OCT findings at baseline (T0)

At T0, all 150 lesions showed the characteristic LC-OCT features of hypertrophic AK. Marked hyperkeratosis with an irregular hyperreflective stratum corneum was present across the entire cohort, consistent with the hypertrophic phenotype required for inclusion. Parakeratosis was a near-universal finding, reflecting the disordered cornification typical of advanced keratinocytic dysplasia. Acanthosis was present in all lesions, with a mean epidermal thickness consistent with values reported for hypertrophic AK in the LC-OCT literature (range 105–126 μm). Keratinocyte atypia of at least moderate degree was documented across the entire cohort, with full-thickness atypia in the 65 KIN 3 lesions (43%). DEJ protrusions consistent with PRO II were identified in 60 lesions (40%) and cone-shaped protrusions consistent with PRO III in 90 lesions (60%). Dilated dermal vessels were observed across all groups, with more prominent and irregular vascular patterns in the KIN 3 subgroup. Intercellular edema was a consistent finding in KIN 3 lesions and was variably present in KIN 2 lesions, reflecting the greater degree of keratinocyte cohesion disruption associated with advanced dysplasia. These baseline differences should be taken into account when interpreting the treatment-specific LC-OCT response patterns described below, as no comparative efficacy analysis was planned.

### 3.3 LC-OCT findings at T1 (24 h post-treatment)

In Cryotherapy group, within 24 h of treatment, all lesions showed a hyporeflective sub-epidermal space on vertical sections, consistent with cryo-induced dermo-epidermal separation. DEJ assessment was not feasible at this time point due to sub-epidermal fluid accumulation. In ablative CO_2_ laser group, the stratum corneum and suprabasal epidermal layers were absent. The papillary dermis was directly exposed, appearing hyperreflective and consistent with early granulation tissue, with dilated vessels visible at the dermal surface. In topical 5-FU/SA group, T1 evaluation was not applicable, as this modality requires continuous daily application; the first post-baseline assessment was therefore performed at T2.

### 3.4 LC-OCT findings at T2 (4 weeks)

In lesions treated with Cryotherapy, blister resolution was documented in all lesions. A marked reduction in hyperkeratosis was observed in the majority of cases; however, residual keratinocyte atypia and DEJ protrusions persisted across the group. All lesions were retreated with a second cryotherapy session. In lesions treated with ablative CO_2_ laser, re-epithelialization was near-complete in all lesions. The neogenerated epidermis appeared thin and regular, with residual atypia limited to isolated basal cells in 35 lesions. Progressive DEJ normalization was already detectable in a proportion of responding lesions. In lesions treated with topical 5-FU/SA group, characteristic features of cytotoxic response were detectable in the majority of lesions, including intraepidermal cystic spaces, prominent longitudinal vessels and perivascular inflammatory infiltrate. A reduction in stratum corneum thickness consistent with progressive keratolysis was observed across the group.

### 3.5 LC-OCT findings at T3 (8 weeks)

In lesions treated with Cryotherapy, LC-OCT-defined complete imaging response was achieved in 60% of lesions. The remaining 40% showed persistent keratinocyte atypia and PRO score elevation and underwent further cryotherapy. Post-inflammatory hyperpigmentation and hypopigmentation were detectable across a substantial proportion of treated lesions at this time point. Complete LC-OCT-defined imaging response was documented in approximately 70% of lesions treated with laser CO_2_. The remaining 30% showed residual LC-OCT features of AK, focal atypia and incomplete DEJ normalization, without clinically apparent disease, and underwent a second CO_2_ laser session, after which complete response was achieved in all cases. In lesions treated with topical 5-fluorouracil/salicylic acid, a heterogeneous response pattern was observed; complete biological response was achieved in a minority of lesions, predominantly KIN 2 / PRO II cases. In the remaining 65% of lesions. predominantly KIN 3 / PRO III, treatment was escalated to a physical modality.

### 3.6 LC-OCT findings at T4 (6–12 months)

At the final follow-up (T4), all lesions fulfilled the predefined LC-OCT criteria for complete imaging response following completion of the treatment strategy adopted during routine clinical management. However, the number of sessions and the pattern of retreatment for the cryotherapy treated lesions was very different from that for the CO_2_ laser treated lesions and from that for the lesions that required change from topical 5-FU/SA monotherapy to a physical modality and which were therefore followed up for the final assessment in the context of the treatment that they had received in the end. The complete LC-OCT-defined imaging responses in the cryotherapy group of patients were achieved by treatment of all of the lesions on multiple occasions and the complete imaging responses were of a quality that was associated with a consistent pattern of long-term scarring and post-inflammatory hypopigmentation in the vast majority of the treated lesions. The complete LC-OCT-defined imaging responses in the CO_2_ laser treated group of patients were of a quality that was associated with a low number of sessions and the results of LC-OCT which in the majority of the treated lesions showed features of dermal neocollagenesis and which also showed that there was either no scarring or that there was minimal scarring in all of the treated lesions. The complete imaging responses in the group of patients with lesions that required change from topical 5-FU/SA monotherapy to a physical modality were of a similar quality to those seen in the other two groups of patients with complete biological responses and in which all of the treated lesions had regressed clinically prior to the final time point and had also regressed on LC-OCT imaging. The three treatment modalities showed distinct longitudinal LC-OCT response patterns, differing in the number of treatment sessions required, the need for retreatment or treatment escalation, and the structural tissue changes observed during follow-up.

## 4 Discussion

This retrospective study supports the role of LC-OCT as a non-invasive tool for monitoring imaging response in hypertrophic actinic keratoses treated with cryotherapy, ablative CO_2_ laser, and topical 5-FU/SA. In contrast to clinical examination and dermoscopy, LC-OCT enabled visualization of residual keratinocyte atypia, dermal-epidermal junction abnormalities, PRO score changes, vascular patterns, and modality-specific post-treatment tissue responses (15, 40, 42, 43).

Among currently available non-invasive skin imaging techniques, LC-OCT occupies an intermediate position by combining near-cellular resolution with sufficient penetration depth to assess the epidermis, dermal-epidermal junction, and superficial dermis, thereby bridging the gap between high-resolution superficial imaging and deeper but lower-resolution modalities.

One of the main observations of this study was that apparent clinical improvement did not always coincide with normalization of all LC-OCT imaging features. In several lesions, persistent keratinocyte atypia, residual DEJ abnormalities, or persistent PRO score alterations remained detectable despite apparent clinical resolution. These findings suggest that LC-OCT may provide additional information beyond conventional clinical assessment during follow-up. This is particularly relevant for hAKs, which often show marked hyperkeratosis and advanced KIN/PRO features associated with increased risk of progression to invasive squamous cell carcinoma. Importantly, a complete LC-OCT imaging response does not exclude residual subclinical disease: given a penetration depth of approximately 500 μm, LC-OCT may fail to capture deeper or focally persistent atypia, which further supports the need for prolonged clinical surveillance and, where indicated, histological correlation.

The three treatment modalities showed distinct longitudinal LC-OCT response patterns rather than directly comparable efficacy profiles. Cryotherapy was characterized by early dermo-epidermal separation and frequently required repeated treatment sessions before imaging normalization was observed. CO_2_ laser was associated with immediate tissue ablation followed by rapid re-epithelialization and characteristic dermal remodeling (46), whereas topical 5-FU/SA produced a more heterogeneous imaging response, particularly in lesions with more advanced KIN and PRO scores. Because treatment allocation was based on routine clinical decision-making rather than randomization, these observations should be interpreted as descriptive treatment-specific response patterns rather than evidence of comparative therapeutic superiority.

These findings suggest that LC-OCT may help guide individualized treatment decisions in hAK by identifying incomplete response and supporting retreatment or treatment escalation before clinical recurrence becomes evident. LC-OCT-derived criteria for complete response may therefore provide a more informative endpoint than clinical clearance alone.

### 4.1 Limitations

Several limitations should be acknowledged. First, the retrospective observational design and clinician-driven treatment allocation introduce an inherent risk of selection bias and preclude comparative efficacy analyses among treatment modalities. Second, histopathological confirmation was not systematically available either at baseline or after treatment, and the imaging-defined response adopted in this study should therefore not be interpreted as histological clearance. Third, LC-OCT image assessment reflected routine clinical practice and was not performed in a blinded fashion, nor was formal interobserver agreement evaluated. Finally, although follow-up extended to 6–12 months, longer prospective studies incorporating standardized imaging protocols, histopathological correlation, and recurrence assessment will be required to validate LC-OCT-derived imaging response criteria. We also acknowledge that, in the absence of histological sampling, some advanced KIN 3 / PRO III lesions could potentially have harbored foci of *in situ* or early invasive squamous cell carcinoma not detected by imaging; LC-OCT should therefore not be regarded as a substitute for histopathology in excluding invasion.

## 5 Conclusion

LC-OCT enables non-invasive imaging-based monitoring of hypertrophic actinic keratoses treated with cryotherapy, ablative CO_2_ laser, and topical 5-FU/SA. By visualizing residual keratinocyte atypia, DEJ abnormalities, PRO score changes, vascular patterns, and modality-specific tissue responses, LC-OCT can detect incomplete clearance that may not be clinically apparent. These findings suggest that LC-OCT may support individualized treatment selection, guide retreatment or escalation strategies, and provide more robust imaging-based endpoints for future studies on actinic keratosis management.

## Data Availability

The raw data supporting the conclusions of this article will be made available by the authors, without undue reservation.
